# Within- and across-breed genomic prediction using whole-genome sequence and single nucleotide polymorphism panels

**DOI:** 10.1186/s12711-016-0193-1

**Published:** 2016-02-19

**Authors:** Oscar O. M. Iheshiulor, John A. Woolliams, Xijiang Yu, Robin Wellmann, Theo H. E. Meuwissen

**Affiliations:** Department of Animal and Aquaculture Sciences, Norwegian University of Life Sciences, 1432 Ås, Norway; The Roslin Institute (Edinburgh), Royal (DICK) School of Veterinary Studies, University of Edinburgh, Midlothian, EH25 9RG Scotland, UK; Institute of Animal Husbandry and Animal Breeding, University of Hohenheim, 70593 Stuttgart, Germany

## Abstract

**Background:**

Currently, genomic prediction in cattle is largely based on panels of about 54k single nucleotide polymorphisms (SNPs). However with the decreasing costs of and current advances in next-generation sequencing technologies, whole-genome sequence (WGS) data on large numbers of individuals is within reach. Availability of such data provides new opportunities for genomic selection, which need to be explored.

**Methods:**

This simulation study investigated how much predictive ability is gained by using WGS data under scenarios with QTL (quantitative trait loci) densities ranging from 45 to 132 QTL/Morgan and heritabilities ranging from 0.07 to 0.30, compared to different SNP densities, with emphasis on divergent dairy cattle breeds with small populations. The relative performances of best linear unbiased prediction (SNP-BLUP) and of a variable selection method with a mixture of two normal distributions (MixP) were also evaluated. Genomic predictions were based on within-population, across-population, and multi-breed reference populations.

**Results:**

The use of WGS data for within-population predictions resulted in small to large increases in accuracy for low to moderately heritable traits. Depending on heritability of the trait, and on SNP and QTL densities, accuracy increased by up to 31 %. The advantage of WGS data was more pronounced (7 to 92 % increase in accuracy depending on trait heritability, SNP and QTL densities, and time of divergence between populations) with a combined reference population and when using MixP. While MixP outperformed SNP-BLUP at 45 QTL/Morgan, SNP-BLUP was as good as MixP when QTL density increased to 132 QTL/Morgan.

**Conclusions:**

Our results show that, genomic predictions in numerically small cattle populations would benefit from a combination of WGS data, a multi-breed reference population, and a variable selection method.

## Background

Genomic selection (GS) is becoming the standard approach to generate genetic progress in livestock. It was pioneered in the dairy cattle sector because of its potential to achieve high accuracy for non-phenotyped animals, thereby reducing generation intervals by reducing the need for progeny-testing. It has been implemented through the use of panels of SNPs (single-nucleotide polymorphisms) that are distributed over the whole genome, and various commercial bovine SNP chips are available, with densities ranging from 3k to 777k [high-density (HD) panels]. So far, results of several GS studies in livestock that have been summarized in [1] show that genomic estimated breeding values (GEBV) can be significantly more accurate than EBV based on phenotypes. Dairy cattle such as the Holstein have greatly benefitted from GS through increases in the accuracy of GEBV resulting from a large reference population and low effective population size (Ne). However, the impact of GS on numerically small breeds, sometimes with larger effective population sizes, is much less significant. This is often exacerbated by the fact that greater emphasis is put on functional traits in these breeds, which typically have lower heritabilities than production traits. Solberg et al. [2] and Su et al. [3] have compared the use of HD to 54k SNP chips and reported small or no increases in accuracy of GEBV or increases for some traits only. Pooling animals from related breeds has been proposed as an option to overcome the small size of reference populations but has not been very successful due to non-persistent associations between SNPs and QTL (quantitative trait loci) across breeds (populations), or inconsistent linkage disequilibrium (LD) between SNPs and QTL across populations [4–6].

As a consequence of these results, progress in the genomic evaluation of dairy cattle has been largely based on the use of 54k SNP panels and primarily restricted to evaluations within breeds. However, new opportunities for GS will arise from the rapid advances in next-generation sequencing technologies e.g. [7], with whole-genome sequence (WGS) data becoming available for large numbers of individuals. Such data need to be explored for their potential in across-breed evaluations. WGS data differ fundamentally from the current data obtained with dense SNP chips because all variants, such as SNPs, indels, copy number variants (CNV), etc., are included. Since all variants, both rare and common, are captured for a population, WGS data could provide more precise signals for causative mutations, both within and across families; hence predictions would no longer have to completely rely on linkage disequilibrium (LD) between SNPs and QTL. Consequently, WGS data could lead to more accurate genomic predictions. In the case of across-breed predictions, the use of WGS data could reduce or remove the need to rely on associations between SNPs and QTL which may not persist across the breeds being evaluated [8].

Although Meuwissen and Goddard [9] reported an advantage of WGS data over dense SNP data using simulated data, their results were restricted to within-population predictions and to a small number of QTL/Morgan. Therefore, the advantages of WGS data for across-breed predictions and divergent small populations remain largely unknown. Hence, the first objective of this study was to assess how much predictive ability is gained by using WGS data under varied QTL densities and trait heritabilities compared to different SNP densities, with emphasis on divergent dairy breeds with small populations and large effective population sizes (>100). Secondly, we assessed the relative performance of the use of a non-variable selection method [SNP-based best linear unbiased prediction (SNP-BLUP)] and a variable selection method (MixP; [10]) for genomic prediction.

## Methods

### Scaling of the simulated populations

GS generally requires large training populations, and thus the simulation and analysis of WGS data (with millions of SNPs), which comprise many large and replicated populations over many generations, are computationally prohibitive. In this study, we followed the scaling argument used and tested by [9, 11], which is based on the equation developed by [12, 13] for expected accuracy of genomic prediction. According to [12, 13], the accuracy of genomic prediction depends on the parameter λ = Th^2^/ML, where T is the number of individuals with genotypes and phenotype in the training data, h^2^ is the heritability of the trait, M is the effective number of loci per Morgan (~2Ne), and L is the genome size in Morgan. If we scale the genome size down from 30 to 1 Morgan, and simultaneously reduce the training population size by a factor of 30, λ remains constant and, thus, the accuracy of prediction will not be affected. E.g. a large-scale simulation with 6000 training animals with a 30-Morgan genome, yields approximately the same accuracy of genomic prediction as a training population of 200 animals with a 1-Morgan genome, which requires less computer resources.

### Simulation of whole-genome sequence data

The parameters used in the simulation of the genome and population structure are summarized in Table 1. The simulation was based on a forward-in-time approach. A Fisher-Wright idealized population was simulated [14], with a mutation rate of 10^−8^ per bp per meiosis, assuming 1 Mb per cM and a historical effective population (Ne) size of 200. The simulation was conducted for a minimum of 1950 or 1990 generations, to create a mutation-drift-recombination equilibrium. Previous studies have shown that this is sufficient to establish equilibrium [15, 16]. At each generation, a breeding pool of 100 males and 100 females was generated, and mating proceeded with random sampling of a sire and a dam for each offspring. Therefore, mutation and drift were the only two evolutionary forces considered.

**Table 1:** Population structure and parameters used in the simulation

Number of chromosomes	1
Genome length	1 Morgan
Mutation rate	10^−8^/bp/meiosis
Effective population size (Ne)	200
Recombination	Haldane map function
QTL density	45 or 132/Morgan
QTL effects	Normal distribution
Number of generations	1950 + 50 or 1990 + 10
Heritability	0.30 and 0.07

To simulate two diverged populations, the population was split into two to represent separate breeding populations, Population A and Population B. This divergence took place after either 1950 or 1990 generations and each of the populations was simulated for a further T generations (T = 50 or 10), such that each population was propagated for 2000 generations. Hence, SNP mutations that occurred after 1950 or 1990 generations were specific to each population. After the divergence, only within-subpopulation matings occurred (i.e. there was no exchange of genetic material between sub-populations), with a Ne of 200 for each sub-population. At generation 2000, census size was increased to 500 individuals in each population. Among these 500 individuals, 200 were randomly sampled and phenotypes were generated to form a reference population for estimation of marker effects, while the remaining 300 individuals were used to form a validation population, for which breeding values were predicted from their genotypes.

### QTL densities and datasets

The mutation-drift process resulted on average in 4648 variants that were distributed across a 1-Morgan chromosome with a minor allele frequency (MAF) higher than 0.02 and a standard deviation of the number of variants of 125. All variants were treated as SNPs. Among the SNPs generated, 45 or 132 loci were randomly sampled and designated as causative QTL, which resulted in 4603 or 4516 remaining SNPs. For each population, there were on average 45 (132) randomly sampled QTL SNPs. All SNPs, including the QTL represented the WGS data. Different SNP densities were then created by randomly sampling without replacement from the non-QTL loci. These panels contained 3000, 2000, 1000, or 200 SNPs and were named data3000, data2000, data1000, or data200, respectively. These densities are equivalent to 90k, 60k, 30k, and 6k SNP panels for a 30-Morgan bovine genome.

### Genetic and phenotypic values

Two traits, with heritabilities of 0.30 and 0.07, were simulated for each scenario. Since for quantitative traits, a large proportion, typically more than half, of the total genetic variance is additive and responsible for most of the genetic variation within a population [14, 17], we assumed only an additive genetic model. An allelic effect ($a_{j}^{{\prime }}$) was assigned to the reference allele (allele “1”) of each QTL by sampling effects from a normal distribution. After sampling, their effects were standardized to achieve a total genetic variance of 1, by $a_{j} = a_{j}^{{\prime }} /\sqrt {\sum\nolimits_{k} {2p_{k} (1 - p_{k} )(a_{k}^{{\prime }} )^{2} } }$, where subscripts *k* and *j* denote the *k*-th and *j*-th QTL, the summation is over all QTL, and *p*_*k*_ is the within-population frequency of allele “1” of the *k*-th QTL. Then, the total genetic value for individual *i* was calculated as:

$$g_{i } = \mathop \sum \limits_{j = 1}^{{N_{QTL} }} x_{ij} a_{j } ,$$

where *x*_*ij*_ is the number of alleles “1” that individual *i* carries at locus *j*. Phenotypes were generated by adding environmental effects drawn from a normal distribution with a mean of zero and variance such that heritability of the trait was 0.30 or 0.07 in generation 2000 within each population.

### Design of evaluations with reference and validation populations

In Scenario 1, predictions were calculated within population A using its own reference population of 200 individuals, with the remaining 300 individuals from the same population used for validation. In Scenario 2, across-population predictions were calculated for population B using the reference population of population A to predict the breeding value of individuals in the validation population of B. In Scenario 3, predictions were based on a multi-breed reference population after combining the reference populations of A and B to reach a total number of 400 individuals. Validation was performed within-breed, using 300 individuals from population A. This Scenario 3 can occur in practice when breeders of populations A and B combine their reference populations, with the aim of increasing accuracy for their own population. In Scenario 4, we checked the impact of increasing the reference population of A in Scenarios 1 and 2 by increasing its size to 400 and validating it by predicting breeding values in either A or B and comparing it to multi-breed estimation of SNP effects.

### Estimation methods and data analysis

Two methods were used to estimate SNP effects in the reference population: SNP-BLUP and MixP [10]. SNP-BLUP estimates the effects of SNPs by best linear unbiased prediction [18] and was implemented using a ridge regression model of the SNP effects that is equivalent to model 2 of VanRaden [19]. MixP is similar to BayesC [20] except that SNPs with small effects are assumed to explain part of the genetic variance instead of having no effect. Therefore, MixP assumes that SNP effects come from a mixture of two normal distributions [10], i.e. one with a large variance ($\sigma_{1}^{2}$) and one with a small variance ($\sigma_{2}^{2}$). The distribution of the total genetic variance (Vg) over the ‘large’ SNPs and the ‘small’ SNPs followed the Pareto principle (hence the P in MixP), such that $x\%$ of the SNPs with the largest effects are responsible for $(100 - x)\%$ of the genetic variance. Given the prior for the mixing frequency $(\pi = x/100)$ and using the Pareto principle, the variances of the large and small SNP effects are respectively:

$$\left. {\begin{array}{*{20}l} {\sigma_{1}^{2} = \frac{{\left( {1 - \pi } \right)V_{g} }}{{\pi N_{m} }}} \hfill \\ {\sigma_{2 }^{2} = \frac{{\pi V_{g} }}{{\left( {1 - \pi } \right)N_{m} }}} \hfill \\ \end{array} } \right\},$$

where $N_{m}$ is the total number of genotyped SNPs, such that $N_{m} (\pi \sigma_{1 }^{2} + \left( {1 - \pi } \right)\sigma_{2}^{2} ) = V_{g}$. The π value used for MixP was set to $N_{QTL} /N_{m}$ (i.e. number of QTL simulated vs. number of SNPs used). A preliminary study on the optimal values of π revealed that values around $N_{QTL} /N_{m}$ were close to optimal and that deviations from this value hardly affected the accuracy of genomic prediction.

The linear model used to estimate SNP effects for both SNP-BLUP and MixP approaches was as follows:

$${\mathbf{y}} =\upmu + \mathop \sum \limits_{j = 1}^{{N_{m} }} \mathbf{X}_{\mathbf{j}} b_{j } + {\mathbf{e}},$$

where **y** is a N × 1 vector of phenotypes; µ is the overall mean; $N_{m }$ is the total number of genotyped SNPs; ${\mathbf{X}}_{{\mathbf{j}}}$ is a N × 1 vector of the N standardized SNP genotypes, i.e. ${\mathbf{X}}_{{\mathbf{j}}} = \frac{{ - 2p_{j} }}{{\sqrt {2p_{j} \left( {1 - p_{j} } \right)} }}, \frac{{1 - 2p_{j} }}{{\sqrt {2p_{j} \left( {1 - p_{j} } \right)} }},\;{\text{or}} \frac{{2\left( {1 - p_{j} } \right)}}{{\sqrt {2p_{j} \left( {1 - p_{j} } \right)} }}$ depending on the genotype of individuals *i* “0 0”, “1 0”, or “1 1”, respectively and $p_{j}$ is the allele frequency of SNP *j*; $b_{j}$ is the effect of the *j*-th SNP genotype; and **e** is a N × 1 vector of environmental effects assumed to be distributed as N(0, ${\mathbf{I}}\sigma_{e}^{2}$). For the SNP-BLUP approach, $b_{j}$ is assumed to follow the distribution N(0, $\sigma_{b}^{2}$), where $\sigma_{b}^{2}$ is the SNP variance $\left( {\sigma_{g}^{2} /N_{m} } \right)$, and for the MixP, each $b_{j}$ is N(0, $\sigma_{1}^{2}$) with probability π, or $b_{j}$ is N(0, $\sigma_{2}^{2}$) with a probability (1 − π). The simulated genetic and environmental variances were used as parameters in SNP-BLUP and MixP. MixP used the Iterative Conditional Expectation (ICE) algorithm of Meuwissen et al. [21]. Full details of MixP are provided in [10].

After estimating SNP effects, the GEBV $(\hat{g}_{i} )$ of the validation individuals (i.e. the individuals having only genotypic records) was predicted as:

$$\hat{g}_{i } = \mathop \sum \limits_{j = 1}^{{N_{m} }} X_{ij} \hat{b}_{j ,}$$

where $X_{ij}$ is the standardized SNP genotype of individual *i* for SNP *j*; and $\hat{b}_{j}$ is the estimate of the SNP effect. The correlation between true ($g_{i}$) and estimated genetic value ($\hat{g}_{i}$) was used as a measure of the accuracy of prediction.

### Replication strategy

Simulation procedures were replicated 30 times. Propagation of populations A and B over 2000 generations was repeated 30 times with T = 10 and 45 QTL, and 30 times with T = 10 and 132 QTL. A further 60 full replications of the populations were carried out with T = 50, equally divided between 45 and 132 QTL. Genomic evaluation procedures were then carried out on each of these 120 replicates. The same 120 replicates were used for h^2^ = 0.07 and 0.30 by resampling the phenotypes. Thus, the results are means of 30 replicates for each T (10 or 50) by QTL number (45 or 132) by heritability (0.07 or 0.30) combination. Standard errors were computed as the standard deviation of the accuracies across the 30 replicates, divided by $\sqrt {30}$.

## Results

### Simulated populations

**Fig. 1:**
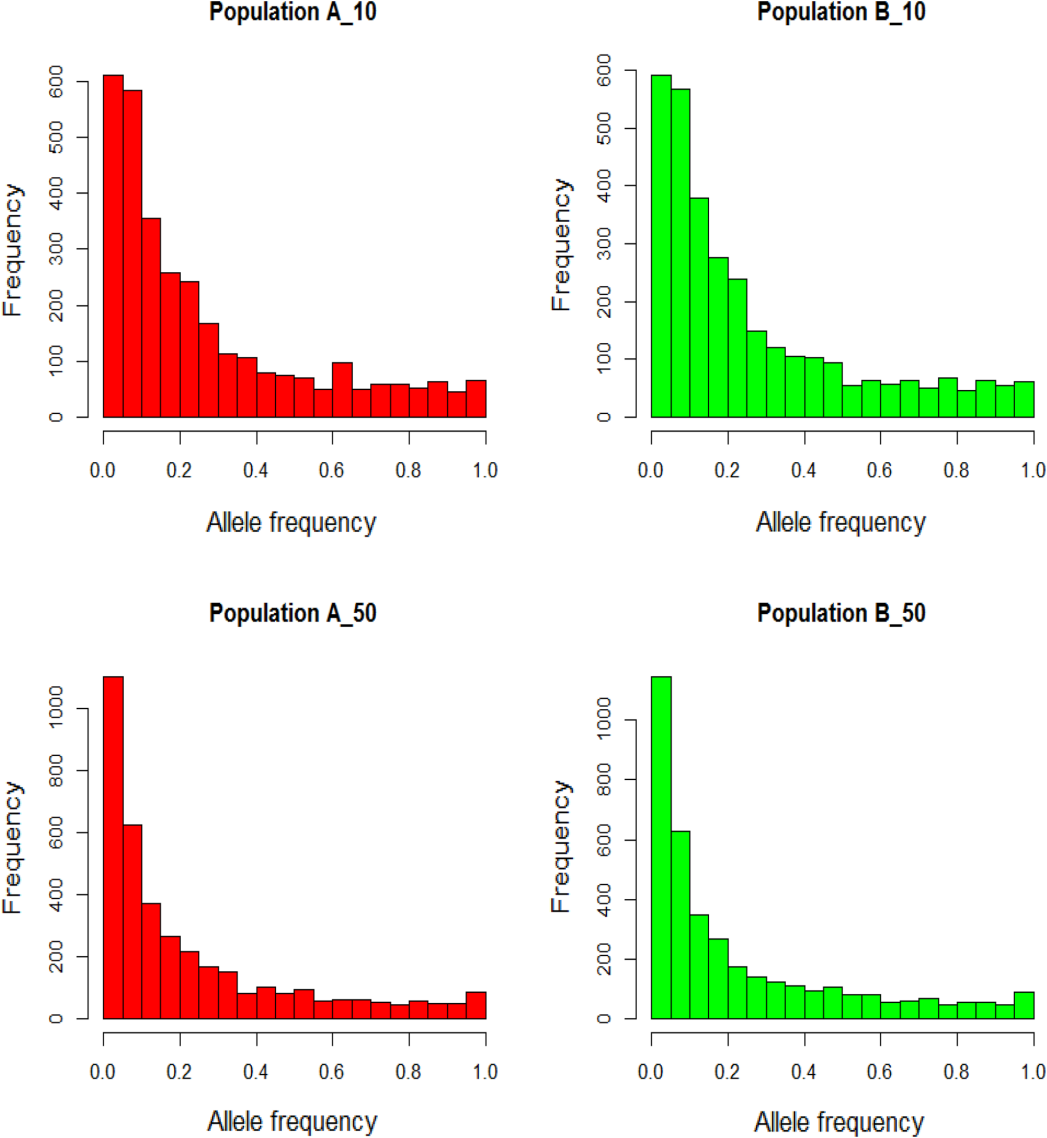
Distribution of allele frequencies in populations A and B at 10 and 50 generations of divergence. A_10 (B_10) and A_50 (B_50) refer to different times of divergence (T = 10 or 50) between both populations. The plots are the result of one replicate. SNP alleles that were fixed in both populations were excluded

**Fig. 2:**
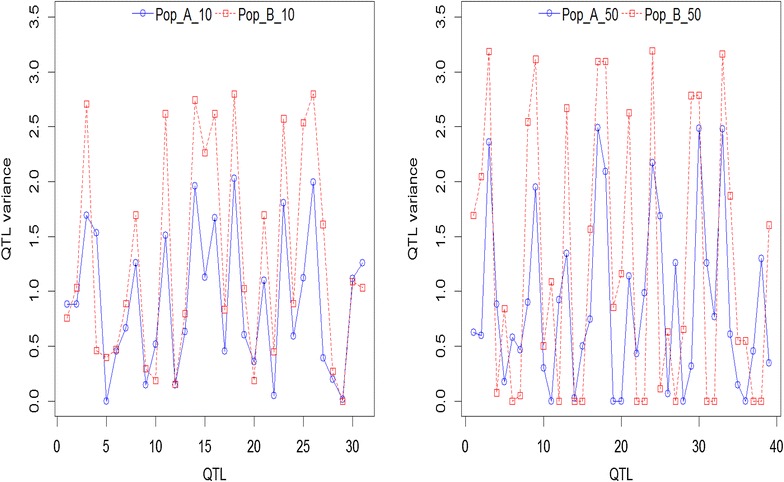
QTL variance for one of the replicates of populations A and B at 10 and 50 generations of divergence. QTL variance was calculated as 2pqa^2^. QTL that were fixed in both populations were excluded. Pop_A and Pop_B refers to populations A and B at T = 10 or 50 generations of divergence, respectively

**Fig. 3:**
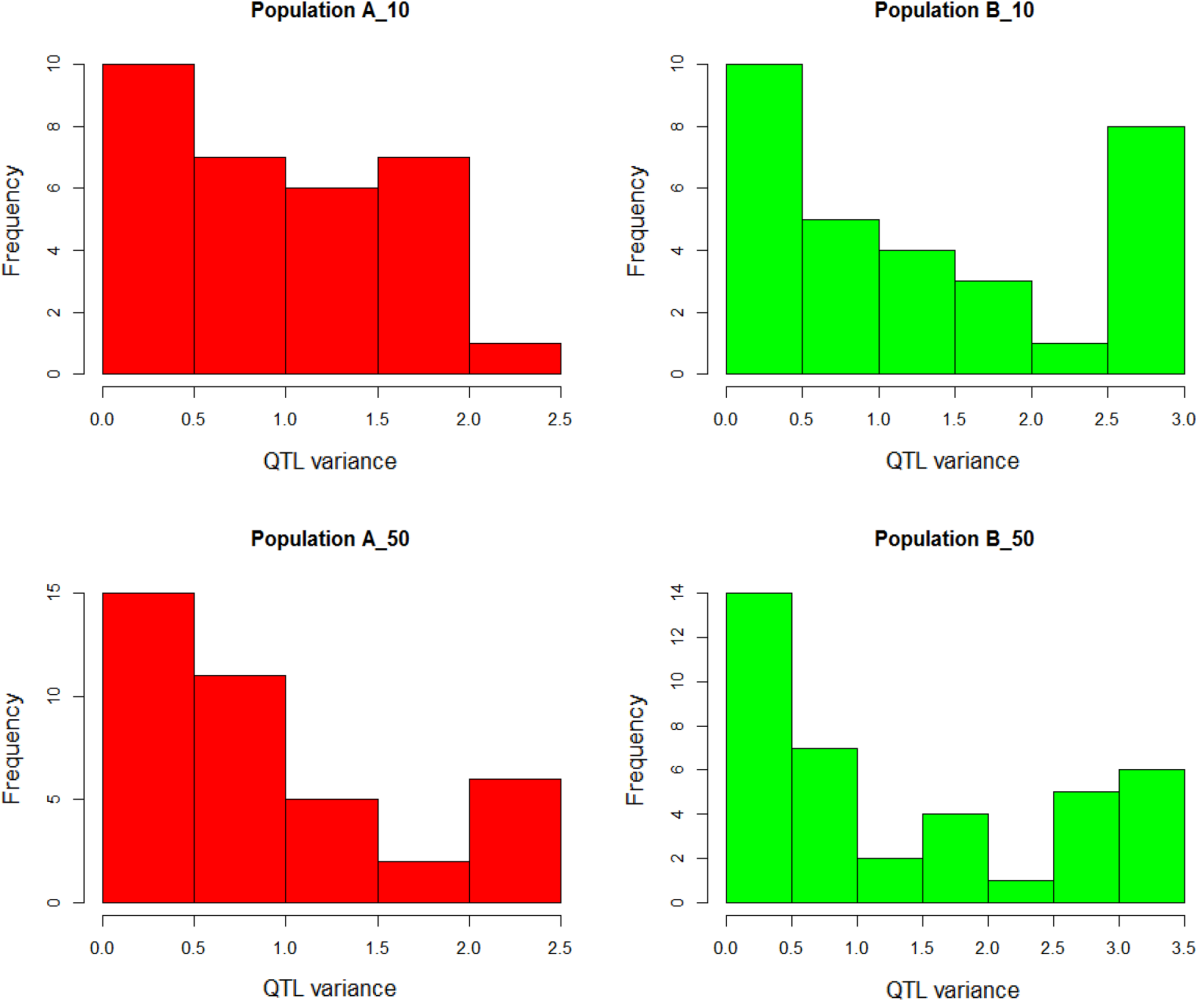
Distribution of QTL variance for populations A and B at 10 and 50 generations of divergence. QTL variance was calculated as 2pqa^2^. A_10 (B_10) and A_50 (B_50) refer to different times of divergence (T = 10 or 50) between both populations. The plots are the result of one replicate. QTL that were fixed in both populations were excluded

**Fig. 4:**
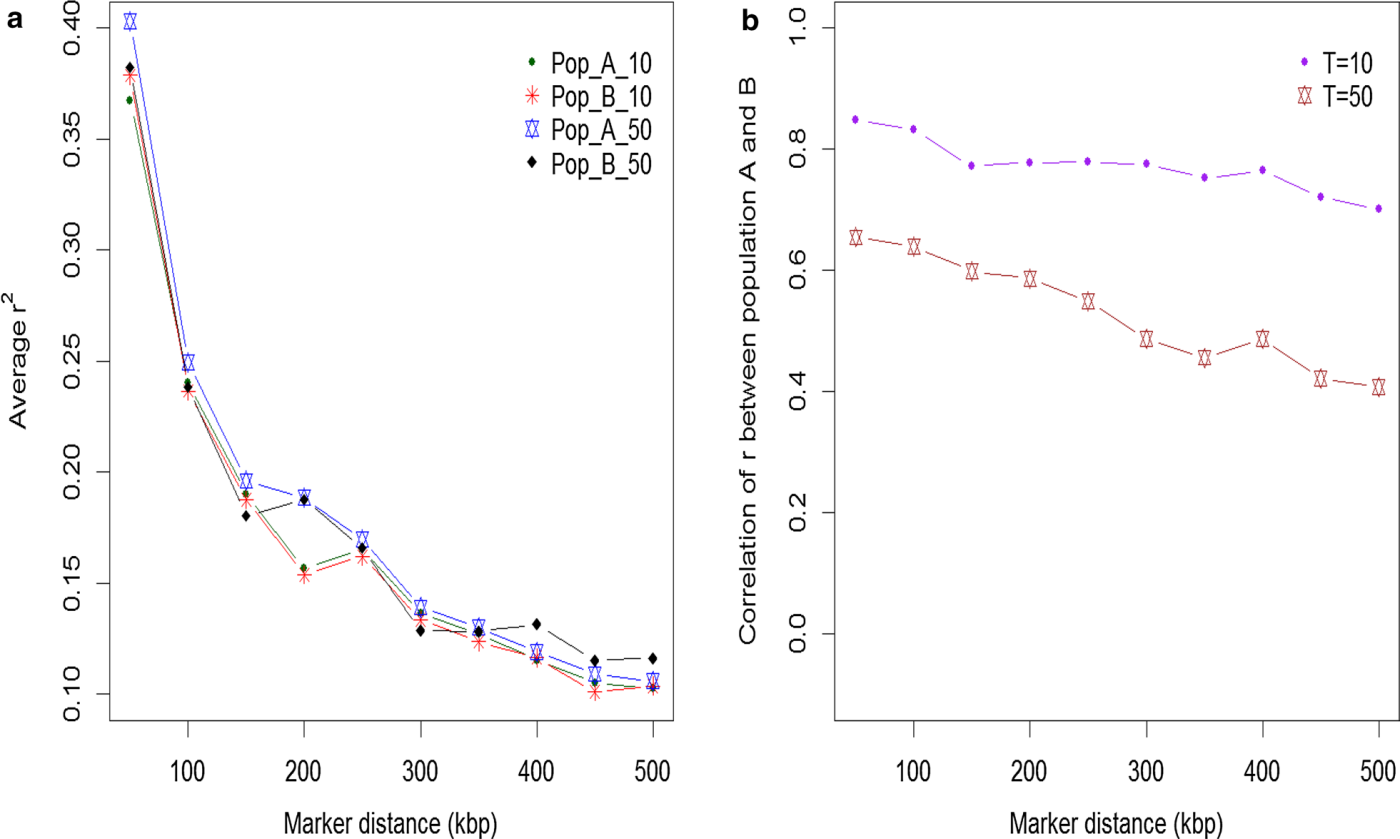
Linkage disequilibrium (r^2^) and persistency of phase (r) as a function of genomic distance. **a** Average linkage disequilibrium (LD) between SNPs estimated according to [39]. Pop_A_10 and Pop_B_10 refer to divergence of populations A and B by 10 generations while _50 refers to divergence by 50 generations. **b** Persistency of LD phase (i.e. the correlation of LD between populations A and B, [40]). Calculations are within populations A and B at different times of divergence (T = 10 or 50). Values are binned at an interval of 50 kb. The plots are the result of one replicate of simulated WGS data. Calculations were done with PLINK [41]

Allele frequencies and QTL variances (2pqa^2^) differed between populations (A and B). As an example, Fig. 1 shows the distribution of allele frequencies for one of the simulated replicates (scenario of 45 QTL/Morgan and T = 10 or 50) for both populations, while Figs. 2 and 3 show the QTL variances. Some SNPs and QTL were fixed in both populations (result not shown). Figure 4 shows the average LD in the WGS dataset, measured as the squared correlation (r^2^) between adjacent SNPs and the persistency of LD phase of adjacent SNPs between the two populations at different times of divergence, measured as the correlation between the two populations of the phased LD, r, of marker pairs. LD ranged from ~0.36 to 0.40 at genomic distances of 0 to 50 kb, respectively, and this trend was similar in both populations. At a genomic distance of 100 kb, LD dropped to about 0.25. As expected, LD decreased further with increasing genomic distance between SNPs (Fig. 4). Persistence of r for adjacent SNPs between populations was equal to ~0.85 at 50 kb for the scenario with T = 10 and ~0.65 for T = 50. This implies that LD of very close SNPs was more persistent between the two populations at T = 10 than at T = 50. A gradual decline in r was observed with increasing genomic distance. For data3000 to data1000, r^2^ and r were lower (especially for data1000) as a result of the decreasing SNP density and increasing inter-SNP distance (result not shown). This also affected r^2^ and r results for data200. The average inter-SNP distances were equal to 21, 33, 50, 100 and 496 kb for the WGS data and data3000 to data200, respectively.

### Scenario 1: Genomic predictions within population A

Accuracies of predictions for population A based on different datasets, trait heritabilities (h^2^), and QTL densities, using SNP-BLUP and MixP are in Tables 2 and 3.

**Table 2:** Accuracy of genomic prediction (±SE) for a trait with a heritability of 0.30 for population A based on SNP-BLUP or MixP using the different datasets

Dataset	45 QTL/Morgan		132 QTL/Morgan	
	Accuracy	% decrease	Accuracy	% decrease
SNP-BLUP				
WGS data	0.596 (±0.015)		0.582 (±0.014)	
data3000	0.578 (±0.016)	3.0	0.575 (±0.014)	1.2
data2000	0.576 (±0.014)	3.4	0.568 (±0.014)	2.4
data1000	0.564 (±0.014)	5.4	0.555 (±0.015)	4.6
data200	0.473 (±0.015)	20.0	0.468 (±0.014)	19.6
MixP				
WGS data	0.632 (±0.018)		0.587 (±0.014)	
data3000	0.598 (±0.018)	5.4	0.579 (±0.014)	1.4
data2000	0.591 (±0.015)	6.5	0.573 (±0.014)	2.4
data1000	0.579 (±0.015)	8.4	0.562 (±0.015)	4.3
data200	0.484 (±0.016)	23.4	0.465 (±0.014)	20.8

Accuracy of prediction was measured as the correlation between simulated true and predicted genetic values in the validation dataset

% decrease in accuracy of prediction relative to that obtained with WGS data

**Table 3:** Accuracy of genomic prediction (±SE) for a trait with a heritability of 0.07 for population A based on SNP-BLUP or MixP using the different datasets

Dataset	45 QTL/Morgan		132 QTL/Morgan	
	Accuracy	% decrease	Accuracy	% decrease
SNP-BLUP				
WGS data	0.413 (±0.024)		0.347 (±0.019)	
data3000	0.400 (±0.023)	3.1	0.332 (±0.019)	4.3
data2000	0.394 (±0.021)	4.6	0.326 (±0.019)	6.1
data1000	0.377 (±0.023)	8.7	0.326 (±0.019)	6.1
data200	0.326 (±0.021)	20.6	0.273 (±0.016)	21.1
MixP				
WGS data	0.431 (±0.028)		0.348 (±0.019)	
data3000	0.407 (±0.025)	5.6	0.333 (±0.019)	4.3
data2000	0.404 (±0.023)	6.3	0.327 (±0.019)	6.0
data1000	0.382 (±0.024)	11.4	0.328 (±0.019)	5.7
data200	0.338 (±0.023)	21.6	0.276 (±0.016)	20.7

Accuracy of prediction was measured as the correlation between simulated true and predicted genetic value in the validation dataset

% decrease in accuracy of prediction relative to that obtained with WGS data

#### Effect of dataset

Use of WGS data resulted in a 1 to 31 % increase in accuracy across the different SNP densities. In all cases, the lowest accuracies were found with the lowest SNP density, i.e. data200, while the highest accuracies were obtained with WGS data. The observed differences in accuracy between the WGS data and data1000 up to data3000 were quite small compared to that between the WGS data and data200.

#### Effect of heritability and QTL density

Accuracy was higher for the trait with a high heritability (0.30) and when QTL density was low. Increasing the QTL density from 45 to 132 per Morgan led to a decrease in accuracy regardless of the heritability of the trait, however, this decrease was greater for the trait with a heritability of 0.07 (Tables 2, 3).

#### Effect of evaluation method

The relative superiority of the two methods depended on QTL density and on trait heritability. At 45 QTL/Morgan, MixP was slightly superior to the SNP-BLUP method, however, the differences between both methods became smaller when the number of QTL increased to 132. The two methods achieved very similar accuracy for the trait with a low heritability (0.07) and 132 QTL/Morgan.

Tables 2 and 3 also show the relative performance of the two methods when using WGS data. Higher accuracies were observed when using MixP, especially at low QTL density. At a density of 45 QTL/Morgan, accuracy increased by 6.0 and 4.4 % with MixP compared to SNP-BLUP for traits with heritabilities of 0.30 and 0.07, respectively, while at a density of 132 QTL/Morgan, accuracy increased only slightly by 0.9 and 0.3 %, respectively. Based on these results, it follows that the predictive ability of MixP decreases as the QTL densities increase, because MixP is not able to fully identify SNPs with larger effects, while the predictive ability of SNP-BLUP is less affected by increasing QTL densities because it assumes that all SNPs have equal variance.

### Scenario 2: Across-population genomic predictions

Table 4 summarizes the accuracies obtained for the different datasets using SNP-BLUP and MixP when the populations have diverged for T generations. The prediction equation from population A was used to estimate GEBV for population B. In summary, Table 4 shows that, for population A, prediction accuracy was significant only when WGS data was used and accuracies were close to zero with the SNP densities.

**Table 4:** Accuracy of across-population genomic prediction (SE) for a trait with a heritability of 0.30 or 0.07 when populations have diverged for T (10 or 50) generations: population A (reference) and population B (validation) based on SNP-BLUP or MixP using the different datasets

Dataset	h^2^ = 0.30				h^2^ = 0.07			
	T = 10		T = 50		T = 10		T = 50	
	SNP-BLUP	MixP	SNP-BLUP	MixP	SNP-BLUP	MixP	SNP-BLUP	MixP
45 QTL/Morgan								
WGS data	0.396 (0.017)	0.482 (0.023)	0.276 (0.021)	0.360 (0.030)	0.270 (0.018)	0.286 (0.022)	0.171 (0.023)	0.197 (0.029)
data3000	−0.004 (0.015)	0.008 (0.016)	0.000 (0.022)	0.026 (0.023)	0.015 (0.016)	0.015 (0.017)	−0.003 (0.019)	−0.003 (0.018)
data2000	0.001 (0.018)	0.001 (0.027)	0.001 (0.017)	−0.002 (0.017)	0.030 (0.018)	0.032 (0.018)	−0.003 (0.017)	−0.017 (0.023)
data1000	0.020 (0.019)	0.009 (0.019)	−0.023 (0.019)	−0.005 (0.020)	−0.013 (0.015)	−0.017 (0.015)	−0.009 (0.014)	−0.005 (0.015)
data200	0.001 (0.013)	−0.010 (0.015)	−0.030 (0.021)	−0.012 (0.017)	0.002 (0.021)	−0.001 (0.022)	−0.034 (0.021)	−0.044 (0.026)
132 QTL/Morgan								
WGS data	0.392 (0.021)	0.403 (0.021)	0.238 (0.019)	0.246 (0.019)	0.209 (0.016)	0.211 (0.015)	0.186 (0.018)	0.186 (0.019)
data3000	0.022 (0.017)	0.025 (0.017)	0.049 (0.023)	0.052 (0.015)	0.005 (0.016)	0.005 (0.017)	−0.018 (0.019)	−0.018 (0.019)
data2000	0.013 (0.017)	0.021 (0.019)	−0.003 (0.017)	−0.004 (0.017)	−0.026 (0.014)	−0.018 (0.012)	0.001 (0.015)	0.001 (0.015)
data1000	0.022 (0.017)	0.028 (0.018)	−0.001 (0.017)	−0.002 (0.017)	0.009 (0.017)	0.009 (0.017)	0.017 (0.018)	0.019 (0.017)
data200	0.005 (0.017)	0.003 (0.015)	−0.022 (0.014)	−0.019 (0.015)	0.002 (0.014)	0.005 (0.017)	−0.005 (0.017)	−0.005 (0.017)

Accuracy of prediction was measured as the correlation between simulated true and predicted genetic value in the validation dataset

At T = 10, prediction accuracies were significantly lower compared to those obtained when reference and validation individuals originated from the same population. Depending on the evaluation method and QTL density, accuracies ranged from 0.39 to 0.48 for a trait with a heritability of 0.30 and when WGS data were used, while accuracies were close to zero when SNPs were used, regardless of their density (Table 4). For the trait with a heritability of 0.07, accuracies ranged from 0.21 to 0.29 when WGS data were used, while accuracies were again close to zero when SNPs were used, regardless of their density.

At T = 50, prediction accuracies were also significantly lower compared to those obtained when reference and validation originated from the same population and at T = 10. Accuracies ranged from 0.24 to 0.36 for a trait with a heritability of 0.30 and from 0.17 to 0.20 for a trait with a heritability of 0.07 when WGS data were used in the different scenarios, while accuracies were close to zero when SNPs were used, regardless of their density. Accuracy was highest (0.36) for a trait with a heritability of 0.30 and when using MixP at a density of 45 QTL/Morgan.

In general, increasing QTL density from 45 to 132 per Morgan, as well as time of divergence from 10 to 50 generations, led to a decrease in accuracy, which was even greater for a trait with a low heritability. MixP was relatively superior to SNP-BLUP when using WGS data in all cases with a density of 45 QTL/Morgan but SNP-BLUP was as good as MixP for greater QTL densities.

### Scenario 3: Multi-breed genomic predictions

Accuracies of multi-breed predictions when populations diverged for T generations are in Table 5. The reference population comprised 200 individuals from each population A and B. Generally, adding 200 individuals from population B to the reference of population A led to a substantial increase in accuracy when using WGS data. Across SNP densities, accuracies were more or less similar to those obtained for within-population predictions (see Tables 2, 3). Prediction accuracies with data200 were always lower than those of the other datasets; a very small increase in accuracy was observed when going from data1000 to data2000 and data3000, while it was much larger with WGS data. The dataset, heritability, QTL density, time of divergence, and evaluation method, all had an effect on the accuracy of prediction.

**Table 5:** Accuracy of genomic prediction (SE) for a trait with a heritability of 0.30 or 0.07 using a multi-breed reference population when populations have diverged for T (10 or 50) generations, based on SNP-BLUP or MixP using the different datasets

Dataset	h^2^ = 0.30				h^2^ = 0.07			
	T = 10		T = 50		T = 10		T = 50	
	SNP-BLUP	MixP	SNP-BLUP	MixP	SNP-BLUP	MixP	SNP-BLUP	MixP
45 QTL/Morgan								
WGS data	0.654 (0.013)	0.710 (0.015)	0.627 (0.013)	0.675 (0.019)	0.475 (0.021)	0.525 (0.025)	0.448 (0.023)	0.480 (0.028)
data3000	0.578 (0.016)	0.602 (0.018)	0.571 (0.016)	0.592 (0.019)	0.404 (0.023)	0.414 (0.024)	0.398 (0.024)	0.409 (0.026)
data2000	0.571 (0.015)	0.574 (0.016)	0.564 (0.016)	0.558 (0.017)	0.400 (0.022)	0.414 (0.025)	0.387 (0.022)	0.390 (0.024)
data1000	0.552 (0.017)	0.551 (0.019)	0.546 (0.017)	0.549 (0.017)	0.376 (0.022)	0.377 (0.023)	0.368 (0.022)	0.367 (0.022)
data200	0.424 (0.017)	0.406 (0.017)	0.428 (0.019)	0.423 (0.021)	0.281 (0.019)	0.273 (0.019)	0.295 (0.020)	0.289 (0.021)
132 QTL/Morgan								
WGS data	0.647 (0.011)	0.662 (0.011)	0.612 (0.014)	0.624 (0.014)	0.410 (0.016)	0.412 (0.017)	0.370 (0.018)	0.374 (0.019)
data3000	0.569 (0.012)	0.573 (0.013)	0.573 (0.015)	0.576 (0.015)	0.343 (0.016)	0.345 (0.016)	0.332 (0.019)	0.332 (0.020)
data2000	0.568 (0.012)	0.570 (0.013)	0.565 (0.015)	0.567 (0.015)	0.341 (0.018)	0.341 (0.018)	0.331 (0.019)	0.330 (0.019)
data1000	0.537 (0.012)	0.536 (0.012)	0.542 (0.016)	0.521 (0.015)	0.313 (0.013)	0.313 (0.013)	0.322 (0.019)	0.321 (0.020)
data200	0.427 (0.017)	0.416 (0.017)	0.430 (0.014)	0.411 (0.014)	0.233 (0.017)	0.224 (0.017)	0.246 (0.016)	0.237 (0.017)

Accuracy of prediction was measured as the correlation between simulated true and predicted genetic value in the validation dataset

At T = 10, a multi-breed reference population using WGS data led to a greater increase in accuracy than that obtained for within- or across-population predictions. Depending on the evaluation method and QTL density, accuracies ranged from 0.65 to 0.71 for a trait with a heritability of 0.30 when WGS data were used, while they ranged from 0.41 to 0.60 when SNPs were used, regardless of their density. For the trait with a heritability of 0.07, accuracies ranged from 0.41 to 0.53 when WGS data were used, while they ranged from 0.22 to 0.41 when SNPs were used, regardless of their density.

At T = 50, a multi-breed reference population using WGS data also led to an increase in accuracy compared to within- or across-population predictions but the increase in accuracy was lower than that obtained at T = 10. With WGS data, accuracies ranged from 0.61 to 0.68 for a trait with a heritability of 0.30 and from 0.37 to 0.48 for a trait with a heritability of 0.07 for the different scenarios. When SNPs at various densities were used, accuracies ranged from 0.41 to 0.59 and from 0.24 to 0.41 for heritabilities of 0.30 and 0.07, respectively.

In summary, increasing QTL density from 45 to 132 per Morgan and time of divergence from 10 to 50 generations led to a decrease in accuracy, although its magnitude depended on trait heritability and the evaluation method. With a density of 45 QTL/Morgan, MixP was superior to SNP-BLUP when using WGS data but SNP-BLUP was as good as MixP when the QTL density increased to 132 QTL/Morgan. Accuracy was highest, i.e. 0.71 for the trait with a heritability of 0.30 at 45 QTL/Morgan and using MixP when populations had diverged for 10 generations.

### Scenario 4: Impact of size of the single-breed versus the multi-breed reference population

Table 6 shows the impact of using either a single- (200 or 400 individuals) or multi-breed reference population on the accuracy of genomic prediction. The impact of increasing the reference population size in the case of across-breed prediction was also analyzed but since the results followed the same trend as those in Table 4, they were not included in Table 6. Multi-breed estimation of SNP effects with WGS data at either 10 or 50 generations of divergence resulted in higher accuracy than when using 200 single-breed individuals. However, single-breed predictions resulted in higher accuracies than multi-breed predictions when equal numbers of reference individuals (400) were used.

**Table 6:** Accuracy of genomic prediction (SE) for a trait with a heritability of 0.30 based on single- and multi-breed reference populations (RP) obtained with SNP-BLUP or MixP using the different datasets

Dataset	Single-breed				Multi-breed			
	RP = 200		RP = 400		RP = 400			
					T = 10		T = 50	
	SNP-BLUP	MixP	SNP-BLUP	MixP	SNP-BLUP	MixP	SNP-BLUP	MixP
45 QTL/Morgan								
WGS data	0.596 (0.015)	0.632 (0.018)	0.696 (0.014)	0.736 (0.017)	0.654 (0.013)	0.710 (0.015)	0.627 (0.013)	0.675 (0.019)
data3000	0.578 (0.016)	0.598 (0.018)	0.681 (0.013)	0.719 (0.015)	0.578 (0.016)	0.602 (0.018)	0.571 (0.016)	0.592 (0.019)
data2000	0.576 (0.014)	0.591 (0.015)	0.679 (0.013)	0.713 (0.014)	0.571 (0.015)	0.574 (0.016)	0.564 (0.016)	0.558 (0.017)
data1000	0.564 (0.014)	0.579 (0.015)	0.668 (0.013)	0.697 (0.014)	0.552 (0.017)	0.551 (0.019)	0.546 (0.017)	0.549 (0.017)
data200	0.473 (0.015)	0.484 (0.016)	0.566 (0.014)	0.576 (0.016)	0.424 (0.017)	0.406 (0.017)	0.428 (0.019)	0.423 (0.021)
132 QTL/Morgan								
WGS data	0.582 (0.014)	0.587 (0.014)	0.691 (0.011)	0.702 (0.012)	0.647 (0.011)	0.662 (0.011)	0.612 (0.014)	0.624 (0.014)
data3000	0.575 (0.014)	0.579 (0.014)	0.681 (0.010)	0.688 (0.011)	0.569 (0.012)	0.573 (0.013)	0.573 (0.015)	0.576 (0.015)
data2000	0.568 (0.014)	0.573 (0.014)	0.670 (0.012)	0.677 (0.012)	0.568 (0.012)	0.570 (0.013)	0.565 (0.015)	0.567 (0.015)
data1000	0.555 (0.015)	0.562 (0.014)	0.654 (0.012)	0.665 (0.012)	0.537 (0.012)	0.536 (0.012)	0.542 (0.016)	0.521 (0.015)
data200	0.468 (0.014)	0.465 (0.014)	0.568 (0.013)	0.563 (0.013)	0.427 (0.017)	0.416 (0.017)	0.430 (0.014)	0.411 (0.014)

Accuracy of prediction was measured as the correlation between simulated true and predicted genetic value in the validation dataset

## Discussion

This study examined the accuracy of genomic prediction for within-population, across-population, and multi-breed reference populations. WGS data, various SNP densities, QTL densities, trait heritabilities, and GEBV estimation methods were used. Results show that the use of WGS data would lead to increased accuracy of genomic prediction for low to moderately heritable traits. Increases in accuracy from using WGS data were much greater with a multi-breed reference population but remained substantial in across-population scenarios. For within-population predictions, the use of WGS data compared to data3000 increased accuracy by 3.1 and 3.3 % (SNP-BLUP) and by 5.7 and 5.9 % (MixP) for traits with heritabilities equal to 0.30 and 0.07, respectively, at 45 QTL/Morgan. With 132 QTL/Morgan, these figures decreased to 1.2 and 4.5 % (SNP-BLUP) and to 1.4 and 4.5 % (MixP). Expanding the reference population with animals from another population (breed) and using WGS data resulted in a remarkable increase in accuracy compared to that achieved by within-population prediction. When SNPs at various densities were used, only a minor or no increase in accuracy was observed. In general, MixP had an advantage over SNP-BLUP at low QTL density, but this advantage decreased as QTL density increased, probably due to the many QTL with smaller effects. With many QTL with smaller effects, the MixP method was less able to pinpoint the SNP(s) that best explained the QTL.

### Within-population genomic predictions

WGS data are not yet available on large numbers of individuals but it should lead to greater accuracies of genomic prediction. Presently, the 1000-bull genomes project [22] makes it possible to impute WGS data on (densely) genotyped animals. It is expected that with WGS data, possibly all variants (including causative mutations) in a population will be captured, which means that genomic prediction does not need to rely completely on LD between SNPs and causative mutations. In all the scenarios investigated, the use of WGS data showed a clear advantage over the use of different SNP densities. Depending on trait heritability, marker density (data1000 to data3000), and QTL density, the observed increase in accuracy when using WGS data for within-population prediction was as high as 13 %. For the lowest SNP density (i.e. data200), the increase reached 24 to 31 %. These results follow the same upward trend in accuracy as observed by Meuwissen and Goddard [9].

In their simulation study, VanRaden et al. [23] excluded the causative mutations but increased the numbers of SNPs from 54k to 500k and reported a gain in accuracy of only 1.6 %. A simulation study by Druet et al. [24] under the neutral model (i.e. when QTL allele frequencies followed the same distribution as other variants in the sequence) found that accuracies increased by only 1.4 % when comparing WGS data with a SNP panel. When the same authors assumed that all the causative mutations had a low MAF, they found that using the WGS data (real/imputed) improved the accuracy of genomic prediction by up to 30 %. Thus, depending on the scenario assumed, there are agreements and differences between our results and those of [24]. These differences in results, especially for the neutral model, may be because Druet et al. [24] used a much denser SNP panel (142,385 SNPs on a 50 Mb genome) and their accuracies were already very high, i.e. around 0.9, which left little room for improvement. On a general note, Druet et al. [24] used a smaller genome (50 Mb with five 10-Mb chromosomes), an effective population size of ~100 and a large reference population of 1021 individuals. The small(er) effective population size leads to extensive LD and a substantially smaller number of effective chromosome segment effects to be estimated, hence better predictions and higher accuracies [25].

In dairy cattle, Hayes et al. [8] used imputed WGS data from the 1000-bull genomes project and observed a 2 % increase in prediction accuracy compared to HD data. Our results differ from those of Hayes et al. [8]. This difference is probably explained by the fact that Hayes et al. [8] used an imputed WGS data, in which case the accuracy of prediction depends in part on how accurately the common and rare variants are imputed. Variants with a MAF higher than 5 % were imputed with an accuracy of about 0.7 to 0.9, while with a MAF lower than 5 %, imputation accuracy rapidly declined [8]. With accurate imputation of common variants, an extra 2 % increase in accuracy of genomic prediction was observed [8], which suggests that if all variants (common and rare) are accurately imputed, a higher accuracy of genomic prediction would be expected with WGS data. As reported by Druet et al. [24], accuracy of genomic prediction could be improved by 2 to 30 % depending on the trait. In inbred *Drosophila melanogaster*, Ober et al. [26] observed no advantage of using WGS data over dense SNP data for genomic prediction. They also reported no difference in prediction performance of SNP-BLUP and BayesB. Their results could be due to: (1) a very large effective population size (~8700), which resulted in a large number of effective chromosome segments (~2000) effects to be estimated; and (2) a small reference population size of about 120. According to [8, 9, 24, 27], the availability of large datasets is important to improve accuracy of genomic prediction even when using sequence data.

In general, the observed increase in accuracy obtained with WGS data can be attributed to the fact that it is not necessary to completely rely on LD between flanking markers and the QTL. However, at a density of 132 QTL/Morgan, MixP and SNP-BLUP performed similarly, which suggests that, in this case, MixP partly relied on LD even when WGS data was used. This means that at high QTL densities (which might be realistic), the ability of MixP to pinpoint the individual QTL with small effects decreases and then relies on LD between SNPs and QTL. According to Meuwissen et al. [18], accuracy of genomic prediction depends on the SNP density and on the LD between SNPs and QTL in order to maximize the proportion of genetic variance explained by the SNPs. However, with WGS data, predictions no longer depend (or at least to a large extent do not) on associations between SNPs and QTL because causative mutations are included in the data and are possibly captured and used in the analysis [27]. Meuwissen and Goddard [9] demonstrated that even with higher SNP densities, an extra gain in accuracy is obtained when including the causative mutations.

### Across-population genomic predictions

Using a reference population (A) to predict GEBV of another population (B) resulted in a significant decrease in accuracies compared to within-population predictions, especially at T = 50 generations of divergence between populations (Table 4). Nonetheless, the across-population accuracies that were obtained using WGS data were substantially higher than with SNP densities, for which accuracies were close or equal to zero. Our results are consistent with the literature [6, 28, 29]. Possible reason(s) for the poor results obtained when SNPs were used regardless of their density could be due to non-persistent associations between SNPs and QTL across populations or inconsistent LD between SNPs and QTL across populations [4–6]. Furthermore, it has been shown that, as the genetic distance between individuals of the reference and validation populations increases, the accuracy of prediction decreases [4, 30–32]. Differences in allele substitution effects between populations result in differences in genetic variance and this could impact predictions across populations [33]. Also, a QTL that segregates in one population may not segregate in the other population, thereby resulting in differences in the genetic variance explained by that QTL between populations (see Figs. 2, 3). The observed differences in QTL variance between populations result directly from differences in allele frequencies, since non-additive effects were not simulated. The use of WGS data suffers much less from changes in LD since it does not need to completely rely on LD between SNPs and QTL. Furthermore, the presence of QTL in the WGS data increases the probability of picking up similar QTL that segregate between populations and that have comparable effects [8]. This may explain why substantially better results were obtained when WGS data was used for across-population prediction, although the accuracies were lower compared to within-population prediction. In summary, the WGS and SNP data differ in the sense that all variants (causative mutations included) are included in the WGS data, which makes it less dependent on LD, while SNP data fully depends on LD.

### Multi-breed genomic predictions

One of the key factors that affects accuracy of genomic prediction is the number of reference animals [6, 25]. Accuracy increases as the number of reference animals increases because the amount of phenotypic data becomes sufficient to detect causative mutations and to distinguish their effects from random noise [27]. Numerically small dairy populations are faced with the problem of a small reference population. Therefore, using a multi-breed reference population could be an option. Our study showed that adding individuals from a second population (population B) to the reference population yielded substantially higher accuracies of GEBV for population A when using WGS data (Table 5). The observed increase in accuracy was greater when the populations had diverged for 10 generations compared to 50 generations, which indicates that relatedness between populations plays a role and should be taken into account when considering a multi-breed reference population for genomic prediction. With the SNP densities that we used, the use of a multi-breed reference population resulted in similar accuracies as those obtained with a single-breed reference population (Table 6). When using equal numbers of reference individuals (400) for multi-breed and single-breed genomic prediction, the single-breed reference population resulted in higher accuracies than the multi-breed reference population (Table 6). Hence, the higher accuracies obtained when using a multi-breed reference population with WGS data can be attributed to: (1) a larger number of reference animals; and (2) the inclusion of causative mutations, which enhances the possibility of picking up similar QTL that segregate between populations and that have comparable effects [8]. The authors of [8] also pointed out that multi-breed prediction using WGS data leads to more accurate predictions because causative mutations that segregate among populations are captured and used in predictions.

According to De Roos [27], the maximum benefit of WGS data can be obtained if the number of reference individuals is increased accordingly. Meuwissen [11] also reported that large reference datasets are needed in order to take full advantage of high-density markers. So far, at least to the best of our knowledge, no study (real or simulation) has evaluated the use of WGS data for multi-breed genomic prediction. However in an imputation study, Bouwman and Veerkamp [34] reported greater imputation accuracy (0.83) when using a multi-breed reference population to impute genotypes from a high-density SNP panel (777k) to WGS, compared to an imputation accuracy of 0.70 when using a single-breed reference population. This shows the benefit of using a multi-breed reference population when reference populations are small. Our study shows that with WGS data and a sufficient number of reference animals, higher genomic prediction accuracies are reached for low to moderately heritable traits.

### Impact of QTL density

In practice, the number of causative mutations that underlie a trait is not known [35]. Thus, we studied two different QTL densities (45 and 132 QTL/Morgan) to investigate the impact of QTL density. We observed that, as the QTL density increased from 45 to 132 (i.e. 1350 or 3960 QTL for a 30-Morgan genome), accuracy decreased markedly. This decrease in accuracy is consistent with results from other simulation studies [9, 10, 25, 35]. As explained by Meuwissen and Goddard, [9], QTL effects and opportunities for their detection become smaller with increasing QTL density, resulting in less accurate GEBV. It should be noted that in spite of the decrease in accuracy when going from 45 to 132 QTL/Morgan, WGS data still resulted in higher accuracies than SNP data regardless of their density.

### Evaluation method

The availability of WGS data for a large number of individuals would provide a large amount of information for genomic prediction. These data would contain millions of variants and it would be necessary to estimate their effects accurately. Therefore, we examined the relative performance of SNP-BLUP and a variable selection method with a mixture of two normal distributions, MixP. The results showed that MixP outperformed SNP-BLUP at a density of 45 QTL/Morgan and also resulted in higher accuracies with WGS data. However, as QTL density increased, accuracy decreased for both methods but MixP still yielded higher accuracies. At a density of 132 QTL/Morgan and for a trait with a low heritability (0.07), both methods gave very similar accuracies, which means that for lowly heritable traits that are controlled by a large number of QTL, SNP-BLUP is as good as MixP. Studies by [9, 10, 25, 35] also demonstrated that when the number of QTL became large, the advantage of allowing for large SNP effects decreased. In such a situation, SNP-BLUP, which assumes a normal distribution with equal variance for all SNP effects, performs as well as variable selection methods. With real data, the performance of these two methods have been reported to be quite similar [3, 36] for most traits. However for traits known to be controlled by a small number of major genes [e.g. *diacylglycerol O*-*acyltransferase 1* (*DGAT1*), which is involved in the control of fat percentage in dairy cattle], Cole et al. [37] and VanRaden et al. [38] reported that variable selection methods outperformed SNP-BLUP. Thus, the method used for genomic prediction is important and the superiority or relative performance of the methods depends on the genetic architecture that underlies the trait [25].

### Assumptions and implications

Using the scaling argument of [9, 11], the results presented here were obtained for scenarios with only one chromosome, and 45 (132) QTL and 4648 variants on average, whereas WGS data in cattle would cover the 30 bovine chromosomes and contain millions of variants and thousands of QTL. The number of SNPs that was simulated (4648 SNPs/Morgan) was much lower than that in real cattle WGS data (~10 to 20 million SNPs/30 Morgan = 0.3 to 0.6 million SNPs/Morgan). This is probably due to the relatively small historical effective population size of 200 used in this study, whereas historical population sizes in cattle were much larger (although current effective sizes are small). This results in a larger number of historical variants that are currently still segregating. An increased number of SNPs to choose from makes it more difficult for variable selection methods to select the right set of SNPs. The small number of variants included in our study made the use of WGS data less challenging for genomic prediction; dealing with millions of variants, as in the case of real WGS data, would be a challenge [8], coupled with an increased number of uninformative variants which might impact accuracy of prediction. Therefore, it would be very beneficial to reduce the amount of uninformative variants as much as possible. Biological information (e.g. coding/regulatory regions and gene sets that are most likely to harbour mutations affecting traits of interest) that is obtained via (1) the analysis of genome annotation and (2) atlases of bovine gene expression, can be used to prioritize and identify a subset of variants that can then be used to impute densely genotyped animals up to sequence and or genomic prediction [8]. In essence, maximizing the accuracy of genomic prediction by using WGS data would very much rely on how well informative variants are exploited and not necessarily on the number of variants.

In our study, we assumed that WGS data contain all causative mutations but that may not be the case in practice, because (rare) SNPs may be missed during (stringent) data filtering or if relatively few individuals are sequenced with limited coverage and the remaining individuals are imputed using SNP chip data. However, for across-breed predictions, the results in Table 4 suggest that it is essential to include the causative mutations in the data and thus (over-) stringent filtering of WGS should be avoided. Finally, when dealing with WGS data, methods that are either able to pinpoint the causative mutations or allow a few variants that are in real high LD to capture the effects of causative mutations and not smear their effects across multiple variants that are in moderate LD with the QTL would be very instrumental to achieve sustained accuracy of genomic prediction across generations [8].

In both the simulation and analysis of data in this study, we assumed only additive genetic effects because it is the most important source of genetic variance and they reflect the actual breeding value of an animal [14, 17]. Furthermore, according to [9], the effects of dominance deviations (the simplest non-additive effects) on the accuracy of genomic prediction using WGS data depend on the QTL i.e.: (1) if there are a few QTL with large effects (3 per Morgan), they are poorly modelled by the additive genomic prediction models; (2) if there are many QTL with small effects (more than 30 per Morgan), the non-additive effects are much smaller and blend with the residual effects, which results in virtually the same accuracy of total genetic value as when gene effects were purely additive (at equal narrow sense heritability).

Although not simulated in this study, dominance and epistatic interactions may result in differences in additive effects between the breeds. If the correlation of the additive effects of QTL between breeds is equal to 0.9 (instead of 1 as assumed here), the accuracy of across-breed genomic prediction would be reduced by 10 %. In the case of multi-breed genomic prediction, the reduction in accuracy of prediction would be less than 10 % (depending on the breed contributions).

We chose to only simulate QTL with a MAF higher than 0.02, which eliminates very rare QTL that may have occurred in only one of the populations, and thus the accuracies of across-breed predictions were favoured. However, very rare QTL would probably not contribute much to the accuracy of prediction, because the genomic prediction models would not detect them.

## Conclusions

This study shows that the use of WGS data can increase accuracy of genomic prediction for low to moderately heritable traits in small populations. This increase in accuracy with WGS data depended on QTL density, the size of the reference population and the evaluation method used. In the absence of a sufficiently large reference population, aggregation of breeds that share close ancestral ties is an option to increase the reference population size and improve accuracy of genomic prediction. The use of WGS data was especially beneficial for multi-breed predictions and when a variable selection method was used. Thus, to take full advantage of a multi-breed reference population, WGS data, large reference sets and variable selection methods are required.
